# A Capsaicin (8%) Patch in the Treatment of Severe Persistent Inguinal Postherniorrhaphy Pain: A Randomized, Double-Blind, Placebo-Controlled Trial

**DOI:** 10.1371/journal.pone.0109144

**Published:** 2014-10-07

**Authors:** Joakim M. Bischoff, Thomas K. Ringsted, Marian Petersen, Claudia Sommer, Nurcan Üçeyler, Mads U. Werner

**Affiliations:** 1 Multidisciplinary Pain Center 7612, Neuroscience Center, Rigshospitalet, Copenhagen University, Copenhagen, Denmark; 2 Department of Neurology, University Hospital of Würzburg, Würzburg, Germany; Glaxo Smith Kline, Denmark

## Abstract

**Background:**

Persistent pain after inguinal herniorrhaphy is a disabling condition with a lack of evidence-based pharmacological treatment options. This randomized placebo-controlled trial investigated the efficacy of a capsaicin 8% cutaneous patch in the treatment of severe persistent inguinal postherniorrhaphy pain.

**Methods:**

Forty-six patients with persistent inguinal postherniorrhaphy pain were randomized to receive either a capsaicin 8% patch or a placebo patch. Pain intensity (Numerical Rating Scale [NRS 0–10]) was evaluated under standardized conditions (at rest, during movement, and during pressure) at baseline and at 1, 2 and 3 months after patch application. Skin punch biopsies for intraepidermal nerve fiber density (IENFD) measurements were taken at baseline and 1 month after patch application. Quantitative sensory testing was performed at baseline and at 1, 2, and 3 months after patch application. The primary outcome was comparisons of summed pain intensity differences (SPIDs) between capsaicin and placebo treatments at 1, 2 and 3 months after patch application (significance level *P*<0.01).

**Results:**

The maximum difference in SPID, between capsaicin and placebo treatments, was observed at 1 month after patch application, but the pain reduction was not significant (NRS, mean difference [95% CI]: 5.0 [0.09 to 9.9]; *P* = 0.046). No differences in SPID between treatments were observed at 2 and 3 months after patch application. Changes in IENFD on the pain side, from baseline to 1 month after patch application, did not differ between capsaicin and placebo treatment: 1.9 [−0.1 to 3.9] and 0.6 [−1.2 to 2.5] fibers/mm, respectively (*P* = 0.32). No significant changes in sensory function, sleep quality or psychological factors were associated with capsaicin patch treatment.

**Conclusions:**

The study did not demonstrate significant differences in pain relief between capsaicin and placebo treatment, although a trend toward pain improvement in capsaicin treated patients was observed 1 month after patch application.

**Trial Registration:**

Clinicaltrialsregister.eu 2012-001540-22 ClinicalTrials.gov NCT01699854

## Introduction

Inguinal herniorrhaphy is a common surgical procedure with more than 800.000 repairs annually in the United States [1]. Persistent severe pain affecting daily activities is present in 5% of patients [2], [3] and in spite of exploratory surgery including selective neurectomy [4], [5], that has been associated with a satisfactory outcome, the invasive nature of the procedure requires availability of medical treatment options for patients reluctant to undergo repeat surgery or not fit for a surgical procedure. The evidence-base for pharmacological treatment of persistent pain following inguinal hernia repair is nearly non-existent and therefore it is of considerable clinical relevance to identify the efficaciousness of drug therapy.

The capsaicin 8% cutaneous patch is used in the treatment of peripheral neuropathic pain and significant pain-relief for up to 12 weeks has been observed in randomized, controlled trials in post-herpetic neuralgia and HIV-related distal neuropathy [6]–[10]. Capsaicin is a selective agonist for the transient receptor potential vanilloid (TRPV1) receptor residing on nociceptive peripheral nerve fibers and keratinocytes [11]. The effect of capsaicin is assumed to be mediated by a reversible defunctionalization of cutaneous nociceptors thereby inhibiting transmission of nociceptive signals [12], [13]. The long lasting pain relieving effect, up to three months after a single capsaicin 8% patch application, is of particular clinical interest. From a pharmacodynamic view, the localized action of the capsaicin patch confers a reduced risk of systemic side effects and a lessened potential for drug interactions. In an un-controlled prospective study (n = 1,044) the capsaicin 8% patch relieved pain and improved sleep quality in patients with various peripheral neuropathic pain states [14]. Interestingly, in this cohort 23% of the patients experienced persistent postsurgical pain of differing etiologies. However, the efficacy of capsaicin 8% patch treatment has never been evaluated in a randomized controlled trial in persistent postsurgical pain. Purified capsaicin has been shown to reduce acute postoperative pain following inguinal hernia repair. In a randomized placebo-controlled trial intraoperative wound instillation of purified capsaicin significantly reduced pain scores in the capsaicin group during the first 4 days after inguinal hernia repair [15].

In the present study we tested the hypothesis that capsaicin 8% patch treatment would be associated with a higher analgesic efficacy compared to placebo in patients suffering from severe persistent inguinal postherniorrhaphy pain.

Since changes in intraepidermal nerve fiber density (IENFD) have been observed following capsaicin 8% patch application in healthy volunteers [16], [17], we investigated the effect of capsaicin on IENFD and sensory function, assessed by thermal and mechanical thresholds, as secondary outcomes. Additional secondary outcomes were changes in ratings of sleep quality, catastrophizing behaviour, anxiety and depression, associated with capsaicin 8% patch treatment.

## Materials and Methods

A randomized, double-blind, placebo-controlled, parallel-group study was conducted at the Multidisciplinary Pain Center, Rigshospitalet, Copenhagen with patients recruitment from August 2012 to May 2013 and follow-up from September 2012 to September 2013. Study approval was obtained from the Committee on Health Research Ethics of the Capital Region of Denmark (H-4-2012-055), the Danish Data Protection Agency, the Danish Medicines Agency (EudraCT-Nr. 2012-001540-22) and the study was registered at ClinicalTrials.gov (NCT01699854). The study was conducted in accordance with Good Clinical Practice (GCP) Guidelines and was monitored by the Copenhagen University Hospital GCP Unit. The protocol for this trial and supporting CONSORT checklist are available as supporting information; see Checklist S1 and Protocol S1.

Patients eligible in the study were referred to the Multidisciplinary Pain Center by a surgeon or a general practitioner and written informed consent was obtained from all patients. Patients were ≥18 years with severe unilateral persistent inguinal postherniorrhaphy pain (numerical rating scale [NRS, 0–10] ≥5) for more than 6 months. Concomitant analgesic medication was allowed if patients had maintained a stable regimen for at least 4 weeks prior to study entry and stayed on stable doses throughout the study period. Exclusion criteria were allergy to capsaicin or vehicle-ingredients in the patch, skin lesions or inflamed skin at the application site, bilateral groin pain, severe cardiac impairment, known diseases impairing nervous system function, alcohol or drug abuse, inability to understand Danish, and in female patients lactation or pregnancy (negative pregnancy test required for females of fertile age).

### Randomization

Randomization was done by Herning Hospital Pharmacy according to a computer-generated randomization list (http://randomization.com/) and a block-size of four was used.

### Pain assessment

Patients evaluated pain intensities (NRS) every morning and evening, at rest in the supine position, during transition from supine to standing position, and during the patient’s palpation of the point of maximum pain in the groin. Assessments were made in the 3 days preceding the patch treatment (baseline) and in the 3 days preceding the clinical visits at 1, 2 and 3 months after patch application.

### Questionnaires

Questionnaires were completed at baseline and at 1, 2 and 3 months after patch application. Anxiety and depression were evaluated with the Hospital Anxiety and Depression Scale (HADS) [18] and the level of catastrophizing behaviour with the Pain Catastrophizing Scale (PCS) [19]. Pain-related sleep interference was evaluated with the Daily Sleep Interference Scale (DSIS [0–10, 0 = pain did not interfere with sleep, 10 = pain completely interfered with sleep]) [20]. The self-report version of the Leeds Assessment of Neuropathic Symptoms and Signs pain scale (S-LANSS) was used for assessment of neuropathic pain components [21].

### Quantitative sensory testing (QST)

**Figure 1:**
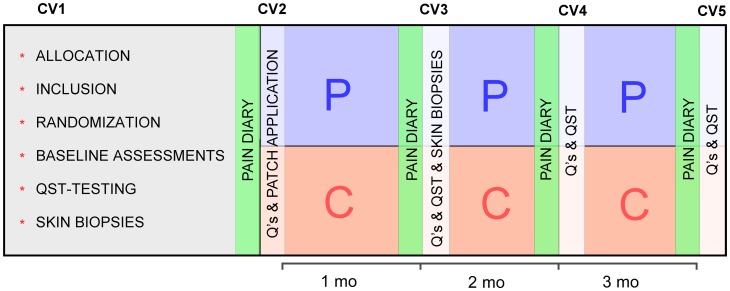
Study algorithm, CV = Clinical visit, Q’s = Questionnaires.

Prior to the QST, hair growth in the inguinal and suprapubic areas was carefully trimmed using a surgical clipper (3M-9671, St. Paul, MN). The QST assessments were performed in accordance with previous studies in persistent inguinal postherniorrhaphy pain [22], [23]. The sensory testing area on the pain side included the point of maximum pain and in addition the contralateral inguinal region was used as a control area. The warmth and cool detection thresholds (WDT, CDT) and the heat pain threshold (HPT) were determined using a computer controlled thermode (Somedic AB, Hörby, Sweden; contact area 2.5×5.0 cm^2^). The baseline temperature was 32°C and thermal stimuli were delivered with a ramp rate of ±1°C/s with cut-off values of 50°C for heat, and 5°C for cold stimuli. The pressure pain threshold (PPT) was assessed at the point of maximum pain using a handheld pressure algometer (Somedic AB, Hörby, Sweden; 1 cm^2^ felt-tipped probe) applied perpendicularly to the skin, until the pressure was perceived as painful or exceeded the cut-off value of 350 kPa. A heat stimulus (5 s at 47°C, ramp rate 1°C/s) was delivered at the point of maximum pain to evaluate the suprathreshold heat pain perception (STH) rated by the patient (NRS). All QST parameters were assessed three times and the median values were used. The QST was performed at baseline and at the clinical visits: at 1 month (day 30–36), 2 months (day 60–66), and 3 months (day 90–96) after patch application (fig. 1).

### Skin biopsies

At baseline and at the clinical visit at 1 month after patch application two 3-mm skin punch biopsies (disposable biopsy punch, Miltex, York, PA) were performed, using a sterile technique, at the point of maximum pain in the groin and on the contralateral control side. Prior to biopsies, the skin areas were anesthetized with 1–2 ml of mepivacaine (10 mg/ml, AstraZeneca AB, Södertälje, Sweden). The IENFD was estimated in agreement with previously described methods [23], [24]. After fixation with 4% paraformaldehyde, the biopsies were washed in phosphate buffer and then stored in 10% sucrose with 0.1 M phosphate buffer at 4°C, until analysis. Sections of 50-µm thickness were immunostained with the pan-neuronal marker PGP-9.5 (1∶800, Ultraclone, Wellow, UK) and visualized with Cy3-coupled anti-rabbit antibodies (1∶100, Amersham Biosciences, Piscataway, NJ). Intraepidermal nerve fiber counting was performed with a light-microscope (Zeiss Axiophot 2, Wetzlar, Germany) and Spot advanced software (Windows Version 4.5, Sterling Heights, MI). The skin biopsies were analyzed blinded in regard to side.

### Treatment procedure and blinding method

The research nurse and the physician responsible for the intervention did only meet the patient on the day of treatment and were not further involved in the study. The research nurse delineated the patch area corresponding to the inguinal pain distribution including the point of maximum pain. Patients were pre-treated with a topical local anesthetic cream (EMLA, lidocaine/prilocaine 25 mg/25 g, AstraZeneca AB, Södertälje, Sweden) 60 min before patch application. The patch application, patch removal, and skin cleansing were done by a physician. During this procedure the research nurse was not allowed presence in the treatment room since minute quantities of aerosolized capsaicin-particles during patch handling could have led to accidental un-blinding of the research nurse.

The capsaicin patches (capsaicin 640 µg/cm^2^, 8% w/w; Astellas Pharma Europe B.V., Leiderdorp, The Netherlands) and the inactive placebo patches were identical in appearance and composition (in regard to vehicle substances). An inactive placebo patch was used since an active placebo patch was not obtainable from the manufacturer of the capsaicin patch. The patients were informed that they might experience pain during and after the patch application. The patches were applied in the groin area for 60 min. After patch removal a cleansing gel (Astellas Pharma Europe B.V., Leiderdorp, The Netherlands) was applied in order to remove capsaicin residues. In order to decrease local irritation of the skin cool packs (assistCo AS, Rjukan, Norway) were administered for 45–60 minutes after patch removal. Patients were told to use oral acetaminophen 1 g every 6 h and ibuprofen 400 mg every 6 h for up to 3 days as needed after patch application. The patients and investigators were blinded to the treatment allocation throughout the study. At the clinical visit 1 month after patch application patients were asked to report if they had experienced application site skin reactions (erythema, pain, burning sensation) or any other adverse events. To evaluate patient blinding we asked patients at the clinical visit 1 month after patch application to verbally report which treatment they had received.

### Statistical analyses

The pain intensities during the 3 standardized conditions (at rest, during movement and pressure evoked), were evaluated twice daily in the 3 days preceding patch treatment (baseline) and in the 3 days preceding the clinical visit at 1, 2 and 3 months after patch application. The median value of the 3 standardized pain assessments was used to calculate the summed pain intensity (SPI) values (containing 6 median values [pain assessments twice daily for 3 days]) in accordance with a previously described method [23]. The summed pain intensity differences (SPID) were calculated for 1, 2 and 3 months after patch application, as the difference between the baseline SPI-value and the post-treatment SPI-value. The primary outcome was comparisons of SPIDs between capsaicin and placebo treatments at 1, 2 and 3 months after patch application. No data from previous studies were available and the *a priori* sample size calculation was therefore based on the best estimates for the study cohort. Based on a significance level of 0.01 (α), a power of 0.9 (β = 0.1), an estimated standard deviation for the SPI assessments of 3.1 (NRS), and a minimal relevant difference for the six SPI assessments of 4.5 (NRS), the estimated number of patients needed in each group were 24 (48 in total). In order to compensate for dropouts the number of patients was set to 50. Due to uncertainties in the power calculation an interim analysis by an independent statistician was planned *a priori* following completion of the first 32 patients.

Linear regression analyses for repeated measures (a mixed model method) were used to evaluate the treatment effects over time. The summed pain intensity difference (SPID) was the outcome variable and treatment group (capsaicin or placebo) and time (1, 2 and 3 months after patch application) were explanatory variables. While measurements taken at different time points were expected to correlate, we also assumed that measurements taken at adjacent time points were more correlated than measurements further apart, and therefore we used an unstructured covariance structure (all covariance parameters are freely estimated). We used the PROC MIXED procedure and the repeated statement in SAS software (9.3, SAS Institute Inc., Cary, NC) to estimate the model. We included the interaction because we were interested in testing whether potential group differences changed over time. The mixed model is dealing with missing longitudinal measures and the analysis included all patients with at least one SPID-value. For secondary outcomes, including QST variables and questionnaire scores (DSIS, HADS, PCS, S-LANNS), differences (Δ) between post-treatment follow-up and baseline values were used to compare capsaicin and placebo patch treatments. The numbers of patients included in the data analyses vary for different outcome measures and are indicated in the tables for each analysis.

The normality of data was assessed with the Kolmogorov-Smirnov test and visual estimation of relevant residual distribution plots. Comparisons between treatment groups were performed with unpaired t-tests (normally distributed data) and the Mann-Whitney tests (non-normally distributed data). Fischer’s exact test was used to compare proportions. Statistical analyses were conducted using SPSS software (SPSS 20.0, Chicago, IL) and MedCalc Software (12.3.0.0, Mariakerke, Belgium). The linear regression analyses for repeated measures were performed with SAS software. Due to the three statistical tests performed (comparisons 1, 2 and 3 months after patch application) and the planned interim analysis, thus increasing the probability of a type I error, all results were considered significant at *P*<0.01 (two-tailed).

## Results

**Figure 2:**
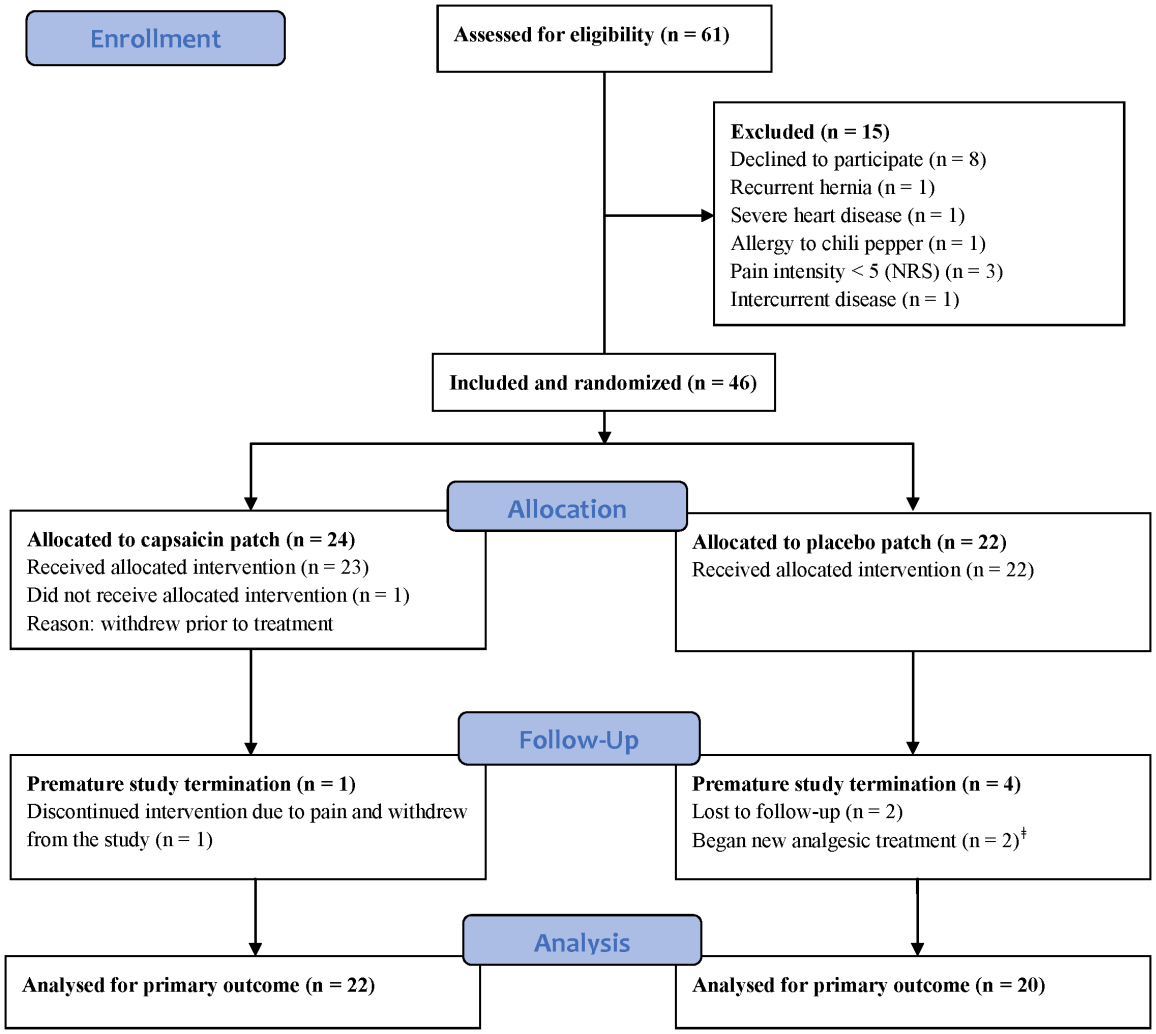
Flow diagram of patients in the study. ^‡^ Data from these two patients were included in the analyses up to the time of the medication violation. NRS = Numerical Rating Scale.

**Figure 3:**
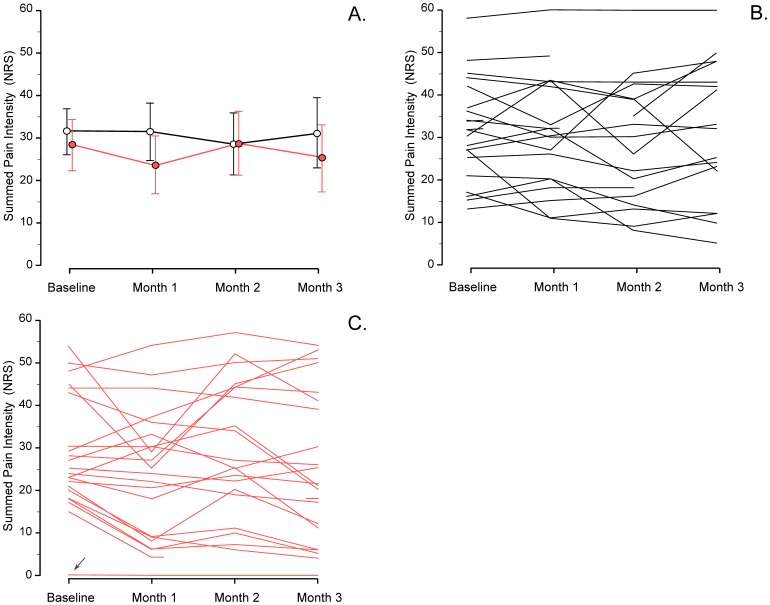
Panel A. Summed pain intensity (SPI) values assessed on a Numerical Rating Scale (NRS) at baseline and at 1, 2 and 3 months after patch application. Each SPI-value contains six median values (pain assessments twice daily for three days) and the maximum SPI-value is 60 NRS units. Values are mean (95% confidence interval). Black line (○) placebo treated patients. Red line (•) capsaicin treated patients. Individual time profiles are displayed for placebo treated patients (panel B, black lines) and capsaicin treated patients (panel C, red lines). The arrow (panel C) indicates patient #10 with baseline median NRS-value of 0 (see text for explanation).

A total of 46 patients were randomized (fig. 2). Baseline patient characteristics and pain ratings are shown in table 1. In one patient (#46) severe pain at the application site necessitated premature patch removal leading to study withdrawal. In two patients (#16, #34) add-on treatment with other analgesics was initiated during the study. Data from these two patients were included in the analyses only up to the time of the violation of the medication criterion. One patient (#10) reported severe pain (NRS ≥5) at the first clinical visit and was thus included in the study in accordance with the protocol. However, in the pain diary evaluations preceding the patch application the patient only reported pain during palpation (NRS = 5), and, no pain (NRS = 0) at rest and during movement Therefore, the median NRS-value of the three standardized pain assessments at baseline was 0 for this patient (indicated in Fig. 3C). Five patients had one or more missing SPID values. Three patients (#4, #15, #49) had one missing SPID value and two patients (#2, #34) had two missing SPID values.

**Table 1:** Baseline patient characteristics.

	Capsaicin (n = 24)	Placebo (n = 22)
Age (yrs)†	52 (17)	55 (14)
Sex (male/female)	20/4	22/0
BMI (kg/m^2^) ‡	25 (23–30)	26 (23–28)
Duration of pain (mo)‡	37 (22–58)	39 (18–63)
Primary/recurrent operation§, *n*	20/4	20/2
Open mesh/laparoscopic§, *n*	21/3	17/5
Unilaterally/bilaterally operated, *n*	23/1	17/5
Exploratory surgery for pain, yes/no, *n*	10/14	7/15
**Concomitant pain medication, yes/no, ** ***n***	11/13	13/9
- Acetaminophen, *n*	7	7
- NSAIDs, *n*	3	7
- Gabapentin, *n*	2	4
- Tricyclic antidepressants, *n*	1	2
- Opioids, *n*	2	2
**Baseline pain ratings** ¶		
- Pain at rest (NRS)	4 (3–7)	4 (3–6)
- Pain during movement (NRS)	5 (4–7)	6 (3–7)
- Pain during palpation (NRS)	7 (5–9)	7 (5–8)

† Mean (standard deviation).

‡ Median (25–75% interquartile range).

§ Pain-generating inguinal hernia operation.

¶ Pain ratings at baseline assessed at rest in the supine position, during transition from supine to standing position, and during the patient’s palpation of the point of maximum pain in the groin. Values are medians (25–75% interquartile range). Two patients who withdrew early from the study and one patient who was lost to follow-up did not report baseline pain ratings and were not included in the analyses of pain ratings.

BMI = Body Mass Index, NRS = Numerical Rating Scale (0–10), NSAIDs = Non-steroidal Anti-inflammatory Drugs.

As previously mentioned, an interim analysis was planned for the first 32 patients, but due to a lower patient-recruitment rate than anticipated and a premature expiry date of the placebo patches (May 2013) an interim analysis could not be performed. The interim analysis would have impeded study progression and prolonged the study for an estimated period of at least three months (time from patch application to study completion; fig. 1). Therefore, it was decided to continue the study without an interim analysis and to recruit patients until the expiration date of the placebo patch: the actual number of randomized patients (n = 46) in the study were thus lower than the originally estimated sample size of 50 patients.

### Pain

The summed pain intensity (SPI) values (NRS) at baseline and at 1, 2 and 3 months after patch application are presented in (fig. 3). A linear regression analysis for repeated measures with SPID as outcome variable and treatment group (capsaicin [n = 22] or placebo [n = 20]) and time (1, 2 and 3 months after patch application) as explanatory variables revealed a significant interaction for group _*_ time (*F*_2,37.4_ = 5.53, *P* = 0.008). The analysis showed that the maximum difference in SPID between capsaicin and placebo treated patients was observed at 1 month after patch application, although statistical significance at the 0.01 level was not achieved. The mean difference [95% CI] in SPID at 1 month after patch application was (5.0 [0.09 to 9.9] NRS-units; *P* = 0.046, table 2), corresponding to a mean difference [95% CI] in SPID percentage of (20.9% [2.9 to 38.9%]; *P* = 0.024). Similarly, there were no significant differences in SPID between capsaicin and placebo patch treatments at 2 months after patch application (mean difference [95% CI]: −1.7 [−6.4 to 3.1] NRS-units; *P* = 0.48) or at 3 months after patch application (mean difference [95% CI]: 3.6 [−3.1 to 10.2] NRS-units; *P* = 0.29).

**Table 2:** Changes in pain intensity between baseline and 1 month after patch application presented as SPID for capsaicin and placebo patch treatments, and the mean difference.

	Capsaicin (n = 22)	Placebo (n = 20)	Difference	*P-*value
SPID (NRS)	4.8 (1.4 to 8.2)	−0.2 (−3.8 to 3.4)	5.0 (0.09 to 9.9)	0.046
SPID %	19.8 (7.5 to 32.1)	−1.0 (−14.1 to 12.1)	20.9 (2.9 to 38.9)	0.024

The SPID was calculated as the difference between the baseline SPI-value and the SPI-value at 1 month after patch application. Each SPI- value contains six median values (pain assessments twice daily for three days). Values are mean (95% CI). Positive values of SPID indicate pain reduction after treatment. *P*-values indicate comparisons of SPID (capsaicin *vs*. placebo).

NRS = Numerical Rating Scale (0–10), SPID = Summed pain intensity difference, SPI = summed pain intensity.

### Sensory Function

Analyses of changes in QST assessments from baseline to 1 month after treatment are presented in table 3. No significant differences between capsaicin and placebo patch treated patients for thermal thresholds, suprathreshold heat pain perception, and pressure pain thresholds were observed.

**Table 3:** Differences in quantitative sensory assessments on the pain side from baseline to 1 month after patch application.

Δ-value	Capsaicin (n = 21)	Placebo (n = 19)	*P-*value
ΔWDT^§^ (°C)	0.2 (−0.9 to 1.2)	0.6 (−0.1 to 1.3)	0.52^‡^
ΔCDT (°C)	0.0 (−0.8 to 0.7)	−0.1 (−0.7 to 0.8)	0.96
ΔHPT (°C)	0.3 (−0.4 to 1.5)	0.3 (−0.2 to 1.2)	0.91
ΔPPT^§^ (kPa)	1.5 (−24.8 to 27.8)	−0.4 (−23.2 to 22.4)	0.91^‡^
ΔSTH (NRS)	0.0 (−1.0 to 0.0)	0.0 (−1.0 to 1.0)	0.56

Values are mean (95% CI)^§^ or median (95% CI). Δ value = post-treatment minus baseline value. *P*-values indicate comparisons of Δ-values (capsaicin *vs*. placebo [unpaired t-test^‡^ or the Mann-Whitney test). Positive Δ-values for WDT, HPT, PPT and CDT indicate increased thresholds after treatment.

WDT = warmth detection threshold, CDT = cool detection threshold, HPT = heat pain threshold, PPT = pressure pain threshold, STH = suprathreshold heat pain perception, NRS = Numerical Rating Scale (0–10).

### Skin innervation

The median (95% CI) IENFD at baseline was significantly lower on the pain side, i.e. 4.3 (1.5 to 6.8) fibers/mm compared with the non-pain side 8.6 (7.4 to 11.0) fibers/mm, (*P*<0.0001, Wilcoxon signed rank test). Changes in IENFD, from baseline to 1 month after patch application on the pain side, did not differ between capsaicin and placebo treated patients (*P* = 0.32, table 4).

**Table 4:** Assessments of intraepidermal nerve fiber density (IENFD) on the pain side at baseline and at 1 month after patch application.

	Baseline(IENFD/mm)	1 month(IENFD/mm)	Δ value	*P*-value
Capsaicin (n = 18)	4.8 (2.5 to 7.2)	2.9 (1.2 to 4.7)	1.9 (−0.1 to 3.9)	0.32
Placebo (n = 20)	5.8 (3.2 to 8.3)	5.2 (2.2 to 8.1)	0.6 (−1.2 to 2.5)	

Values are mean (95% CI). The *P*-value indicates comparison of Δ values (capsaicin *vs*. placebo unpaired t-test). Δ value = baseline –1 month value. Δ values are normally distributed while baseline and 1 month IENFD values are non-normally distributed.

### S-LANSS, Sleep Quality and Psychological Factors

At baseline the median (95% CI) S-LANSS score was 15 (10 to 17) and 26 of 42 patients (62%) reported a S-LANSS score of ≥12 indicating pain components of predominantly neuropathic origin [21]. Changes from baseline to 1 month after patch application with regard to sleep quality, PCS, S-LANSS, and HADS scores did not differ between capsaicin and placebo treated patients.

### Adverse Events

Seventeen of 23 (74%) capsaicin treated patients and 6 of 20 (30%) placebo treated patients reported one or more skin reactions on the application site (*P* = 0.006, Fischer’s exact test, table 5). No other adverse events or complications were observed in the study.

**Table 5:** Skin reactions at application site.

	Capsaicin (n = 23)	Placebo (n = 20)
Any application site reaction, *n* (%)	17 (74)	6 (30)
- Erythema, *n*	9	3
- Pain, *n*	12	6
- Burning sensation, *n*	12	1

### Patient Blinding

Sixteen of 23 patients (70%) treated with the capsaicin patch and 17 of 21 patients (81%) treated with the placebo patch correctly identified their treatment allocation (*P* = 0.49, Fischer’s exact test).

## Discussion

The present study in patients with severe persistent inguinal postherniorrhaphy pain, comparing the pain relief of a capsaicin 8% patch with an inactive placebo patch, was not able to demonstrate significant differences in pain reduction during standardized testing conditions. In addition, we did not observe any changes in secondary outcome variables including sensory function, IENFD, sleep quality, catastrophizing behaviour (PCS), or anxiety and depression (HADS) scores associated with capsaicin patch treatment.

### Statistical Issues

Interpretation of the study’s main finding should consider the *a priori* assigned significance level of 0.01. The authors regarded this conservative statistical measure a precaution against the introduction of type I errors due to the use of multiple comparisons and interim analysis. However, the statistical comparisons of SPID-scores, one month after patch application, yielding *P*-values of 0.024 and 0.046, indicated a trend of an improved pain relief in capsaicin treated patients. A potential contributing factor to the non-significant findings is obviously the premature discontinuation of the study after 46 randomized patients compared to the *a priori* estimated sample size of 50 patients. This circumstance may have introduced a type II error. A post hoc power calculation could be considered but is generally not recommended [25].

### Skin innervation

The study corroborates our previous findings of a reduced IENFD on the pain side compared to the contralateral side in persistent inguinal postherniorrhaphy pain patients, most likely caused by surgical nerve injury [23], [26]. Several studies in healthy volunteers have demonstrated a 60–80% reduction in IENFD one week after capsaicin 8% patch treatment [16], [17], [27]. The current study, the first to evaluate changes in IENFD following capsaicin patch treatment in patients, failed to show a significant difference in IENFD between treatment groups. A plausible explanation is the low baseline IENFD on the pain side in postherniorrhaphy pain patients compared to healthy volunteers, making it difficult to demonstrate a further reduction in IENFD following capsaicin treatment. The small group size may be another reason why the observed reduction of IENFD in the capsaicin group did not reach significance. However, it should be emphasized that none of the studies investigating IENFD after application of an 8% capsaicin patch in healthy volunteers provide data at 4 weeks after application [16], [17], [27], and it cannot be excluded that partial regeneration of nerve fibers already may have taken place [28].

### Sensory Assessments

The QST assessment did not demonstrate any differences in cutaneous (thermal) or deep tissue (mechanical) sensory function between capsaicin and placebo treatment. A possible explanation is the previously mentioned lack of a significant reduction in IENFD after capsaicin treatment. In addition, sensory nerve endings not expressing TRPV1 receptors, i.e. Aβ-fibers, the majority of Aδ-fibers, and a subgroup of C-fibers, remain intact after capsaicin treatment and are thus still capable of transmitting sensory stimuli [12]. Accordingly, previous studies in neuropathic pain patients have failed to demonstrate sensory impairment after capsaicin patch treatment. In painful HIV-related neuropathy no differences in thermal and vibration detection thresholds were observed between capsaicin treated patients and controls at 4 and 12 weeks after patch application [9]. Furthermore, no differences between treatment groups were found in two studies investigating detection thresholds for brush, punctate stimulation, vibration and warmth in patients with post-herpetic neuralgia at 4 and 12 weeks after patch application [8], [29].

### Limitations

A confounding factor for the study is the patient-blinding. The higher incidence of local side effects experienced by patients in the capsaicin group (74%) compared to the placebo group (30%), and the correspondingly high proportion of patients who correctly identified their treatment allocation, indicate an insufficient blinding-technique. Therefore, it cannot be excluded that the potential analgesic effect experienced by the capsaicin treatment may in fact represent a placebo effect. The short duration of the potential analgesic effect (i.e. one month) also may connote to a placebo effect. The study thus supports the use of an active placebo (e.g. low-dose of capsaicin) in order to ensure adequate blinding in future studies assessing the effects of topical capsaicin.

### Pathophysiological Mechanism

Pain relief up to 12 weeks after capsaicin patch application has been observed in previous randomized, controlled trials in post-herpetic neuralgia and HIV-related distal neuropathy [6]–[10]. The lack of a significant analgesic effect in the present study may be explained by a different pain mechanism in persistent inguinal postherniorrhaphy pain. The S-LANNS findings in the present study and the QST results in previous studies [30], [31] point towards neuropathic pain components in this patient cohort, which is also underscored by the reduced IENFD on the painful side, specifying a small-fiber neuropathy. However, a number of postherniorrhaphy pain patients demonstrate decreased pain thresholds to pressure algometry indicating increased deep tissue sensitivity and suggesting deep pain components that may be caused by an untoward inflammatory reaction to the implanted mesh [31]. The negative findings in our study suggest that interruption of cutaneous nerve transmission alone may be insufficient to relieve pain substantially in these patients and seem to indicate that nerve injury, as well as deep tissue inflammation, may contribute to the development and maintenance of persistent postherniorrhaphy pain. Further studies are needed to elucidate the pathophysiological contributors in persistent postherniorrhaphy pain in order to guide future treatment strategies.

In conclusion capsaicin patch 8% did not significantly reduce combined static and dynamic pain components compared to placebo in persistent inguinal postherniorrhaphy pain, albeit a trend towards pain relief was observed 1 month after capsaicin patch application.

## Supporting Information

Checklist S1 **CONSORT Checklist.**
(DOC)

Protocol S1 **Trial Protocol.**
(PDF)
