# Supplementary material for: A Capsaicin (8%) Patch in the Treatment of Severe Persistent Inguinal Postherniorrhaphy Pain: A Randomized, Double-Blind, Placebo-Controlled Trial
Source: PLoS One. 2014 Oct 7;9(10):e109144. doi: 10.1371/journal.pone.0109144 (PMC4188585; doi:10.1371/journal.pone.0109144)
Supplement: Protocol S1 — Trial Protocol. (PDF) [file pone.0109144.s002.pdf]

Indholdsfortegnelse

1. Kontaktpersoner
2. Formål med undersøgelsen
3. Baggrund
4. Hypotese
5. Design
6. Patientpopulation
7. Metode
    a. Smertescore og søvn
    b. Kvantitativ testning
    c. Hudbiopsi
    d. Randomisering og blinding
    e. Undersøgelsesmedicin
8. Outcome
9. Statistik
10. Ulemper for patienten
11. Bivirkninger og komplikationer
12. Respekt for deltagernes integritet
13. Dataregistrering
14. Kvalitetskontrol og kvalitetssikring
15. Indhentelse af samtykke
16. Økonomiske forhold og forsikring
17. Etik
18. Publikation

Capsaicin plaster 8 % til behandling af kroniske smerter efter lyskebrokkirurgi

1. Kontaktpersoner

Koordinerende investigator

Joakim Bischoff, læge, klinisk assistent

Enhed for Kirurgisk Patofysiologi, Afsnit 4114, Blegdamsvej 9, 2100 Kbh. Ø

Tværfagligt Smertecenter, afsnit 7612, Blegdamsvej 9, 2100 Kbh. Ø

tlf. 35 45 76 18

Forsøgsansvarlig investigator og sponsor

Mads U. Werner, overlæge, med.dr.

Tværfagligt Smertecenter, afsnit 7612, Blegdamsvej 9, 2100 Kbh. Ø

tlf. 28 25 77 03

Investigator

Thomas K. Ringsted, forskningssygeplejerske

Tværfagligt Smertecenter, afsnit 7612, Blegdamsvej 9, 2100 Kbh. Ø

tlf. 35 45 76 18

Investigator

Henrik Kehlet, professor, dr. med

Enhed for Kirurgisk Patofysiologi, JMC 4074, Blegdamsvej 9, 2100 Kbh. Ø

tlf. 35 45 12 29

Monitor

Københavns Universitetshospitals GCP-enhed

Bispebjerg Hospital, Bygning 51, 3. sal

Bispebjerg Bakke 23

2400 København NV

Tlf.: 35 31 38 90 Fax: 35 31 38 89

Forsøget udføres i overensstemmelse med protokollen, GCP samt gældende myndighedskrav / lovgivning på området.

Dato og signatur forsøgsansvarlig/sponsor: ____________________

Dato og signatur koordinerende investigator: ____________________

2. Formål med undersøgelsen

Undersøgelsen har til formål at undersøge om capsaicinplaster 8 % (Qutenza) kan reducere kroniske smerter i lysken efter lyskebrokoperation samt at undersøge om der er forskel i virkningseffekt af capsaicinplaster hos patienter med forskel i sensorisk funktion i huden. Vi undersøger også om der ved analyse af hudbiopsi fra lyskerne er forskel i profilen af intraepidermal nervefiberdensitet (IENFD) mellem patienter der responderer på capsaicinplaster sammenlignet med patienter der ikke responderer på capsaicinplaster, hvilket kan bruges fremadrettet ved udvælgelse af patienter til behandling med capsaicinplaster. Endelig vil hudbiopsi med bestemmelse af intraepidermal nervefiberdensitet (IENFD) give histologisk information der kan øge den patofysiologiske forståelse af kroniske smerter efter lyskebrokkirurgi.

3. Baggrund

Der foretages årligt 10.000 lyskebrok operationer i Danmark, de fleste med åben kirurgisk teknik (80 %). Risikoen for kroniske smerter efter lyskebrokkirurgi er 5-12 %.[1] Hos 4-8 % af disse patienter ses invaliderende smerter med kraftige funktionsindskrænkninger der påvirker daglige sociale og arbejdsmæssige aktiviteter.[2] Samfundsøkonomisk har dette betydning, da lyskebrokoperation er en af de hyppigste operationer der foretages i Danmark.
Enhed for Kirurgisk Patofysiologi og Tværfagligt Smertecenter, Rigshospitalet har siden 1. september 2009 haft et multidisciplinært samarbejde omkring patienter med svære kronisk smerter efter lyskebrokkirurgi og vi får henvist patienter fra hele landet. På grundlag af anamnese, klinisk og detaljeret neurofysiologisk undersøgelse kan vi inddele patienterne i formodet inflammatorisk (meche-betinget), neuropatisk (påvirkning af en eller flere af nerverne i lyskeregionen) eller blandet genese til smerterne.[3] Vores gruppe har for nylig på grundlag af de detaljerede præoperative

undersøgelser præsenteret data fra 21 patienter med kroniske smerter efter lyskebrokkirurgi der gennemgik eksplorativ reoperation,[3] hvor der blev foretaget fjernelse af mechen og neurektomi. Patienterne blev fulgt før og efter operationen med målinger af smerte-relateret funktionspåvirkning (AAS [activities assessment scale]).[4] Der sås en statistisk signifikant og klinisk relevant forbedret funktionsscore hos 13 patienter (62 %), uændret funktion hos 5 patienter (24 %) og en forværring i score hos 3 af patienterne (14 %) ved den 6 måneders postoperative kontrol. Der blev desuden observeret postoperative ændringer af de sensoriske forstyrrelser i hud og dybere lag hos en del af patienterne, hen mod normalområdet. Disse behandlingsresultater er internationalt set meget opmuntrende men vi ønsker at tilbyde andre og forbedrede behandlingsmetoder til patienter der ikke ønsker eller egner sig til reoperation, samt til patienter der ikke har haft gavn af reoperation. Det er vanskeligt at behandle neuropatiske smerter farmakologisk, og de midler der benyttes til systemisk behandling, for eksempel tricykliske antidepressiva og antiepileptika, har en del bivirkninger. Capsaicin plaster er et kutanplaster der er godkendt af Sundhedsstyrelsen til behandling af perifere neuropatiske smerter. Capsaicin der findes i chilipebre, er en selektiv agonist for TRPV1-receptoren (transient receptor potential vanilloid 1). Capsaicin stimulerer TRPV1-receptoren, der findes i nociceptorer (smertereceptorer) i huden. Capsaicin plaster indeholder store doser af capsaicin, som frigives hurtigt og overstimulerer TRPV1-receptorerne, hvilket gør dem 'desensibiliserede', så de ikke længere er i stand til at reagere på de stimuli, som normalt forårsager smerte hos patienter med perifere neuropatiske smerter. Capsaicin plasters effekt er ikke tidligere videnskabeligt undersøgt hos patienter med vedvarende smerter efter kirurgi. Derfor vil det være relevant at undersøge om patienter med kroniske smerter efter lyskebrokkirurgi vil have smertelindring med capsaicinplaster, da det vil være et stort behandlingsfremskridt hvis det viser sig smerterne kan behandles lokalt.

I vores patientpopulation er påvirkningen af den sensoriske funktion i smerteområdet i lysken heterogen. Der er således patienter med nedsat sensorisk funktion i området, men også patienter med normal eller forøget sensorisk funktion i smerteområdet.[5] I et tidligere studie med postherpetisk neuralgi patienter tydede det på, at en anden lokalbehandling (lidokain plaster) har større effekt hos patienter med nedsat sensorisk funktion i smerteområdet, når man sammenlignede med en gruppe med normal eller forøget sensorisk funktion i området.[6] For at undersøge om dette også kunne være tilfældet hos patienter med kroniske smerter efter lyskebrok kirurgi, inddeler vi patienterne i to subgrupper på baggrund af neurofysiologisk undersøgelse med kvantitativ sensorisk testning (QST).

Hudbiopsi er en internationalt velaccepteret metode til at karakterisere patienter med kroniske neuropatiske smerter.[7] Hudbiopsi med estimering af intraepidermal nerve fiber densitet (IENFD) kan bidrage med værdifuld information til vores QST målinger.[8] Ved at tage hudbiopsier før og efter behandling i denne undersøgelse kan vi yderligere karakterisere patienterne med henblik på at optimere indikationen for capsaicinplaster. Patienter med kutan hypoæstesi har sandsynligvis en speciel IENFD profil[6] og det er muligt at patienter der responderer på capsaicinplaster har en IENFD profil der er forskellig fra patienter der ikke responderer på capsaicinplaster.[9] Dette kan muligvis bruges fremadrettet til udvælgelse af patienter til behandling med capsaicinplaster.

4. Hypotese

Vores hypotese er at capsaicinplaster kan reducere smerterne hos patienter med kroniske smerter efter lyskebrokoperation, og at der kan være en forskel i virkningseffekt afhængig af om patienterne, vurderet ved QST, har nedsat eller normal/forøget thermal sensorisk funktion i smerteområdet. Desuden er det vores hypotese at patienter der responderer på behandling med capsaicinplaster har en IENFD profil der er forskellig fra patienter der ikke responderer på capsaicinplaster.

5. Design

Undersøgelsen er en randomiseret, dobbeltblindet, placebokontrolleret, parallelgruppe undersøgelse med en opfølgningsfase på 3 måneder efter plasterbehandling. Undersøgelsen forventes påbegyndt 1. august 2012 og forventet afslutning er 1. august 2014.

6. Populationer

En patient, der har gennemført undersøgelsen, er en patient, der har fulgt forsøgets behandlingsplan (per-protokol). En patient, der ikke har gennemført forsøget, er en patient, som inkluderes i forsøget, dvs. giver informeret samtykke og ikke gennemfører forsøget, hvad enten patienten har modtaget forsøgsplastre eller ej (intention-to-treat). Har en patient ikke gennemført forsøget skal der redegøres for om, eller hvorledes denne følges i øvrigt i studiet, - dette gælder også dropouts - samt hvilke data der indsamles fra disse forsøgspersoner. En patient kan tages ud af forsøget hvis investigator skønner, at en ændring af behandlingen vil være det bedste for patienten samt hvis der er sket en overtrædelse af protokollens regler. I overensstemmelse med Helsinki Deklarationen har

patienterne ret til at afbryde forsøget på ethvert tidspunkt af hvilken som helst årsag. Årsagen til at en patient tages ud af forsøget før planlagt, skal noteres i patientens Case Report Form.

6.a Patientpopulation

6.a.1 Inklusionskriterier

- Patienter der har smerter i lysken 6 måneder efter lyskebrok kirurgi (laparoskopisk kirurgi inklusive) med kraftige smerter ≥ 5 (numerisk rating scale 0-10 [NRS]) i hvile eller ved bevægelse. Bilateralt opererede patienter med ensidige smerter kan inkluderes.
- Alder > 18 år
- ASA score I-III
- Patienten har forstået undersøgelsen og har underskrevet informeret samtykke
- Patienten må indtage per orale smertestillende midler under studiet forudsat af at behandlingen har været stabil i mindst 4 uger før forsøgsstart.
- For kvinder i den fertile alder kræves en negativ graviditetstest forud for indgåelse i forsøget og der kræves brug af sikker antikonception (p-piller eller IUD) i 30 dage efter plasterbehandling.

6.a.2 Eksklusionskriterier

- Kendt allergi overfor capsaicin samt plaster.
- Svær hjertesygdom (NYHA ≥ III)
- Umyndig
- Kvinder der ammer
- Forstår ikke dansk i tale eller skrift
- Reducerede kognitive funktioner som forhindrer deltagelse
- Recidiv hernie
- Kendt nervepåvirkning i det pågældende område af anden årsag, fx. følger efter apopleksi, dissemineret sklerose, diskusprolaps osv.
- Alkohol/medicinmisbrug.
- Kan ikke kooperere til QST-undersøgelse
- Bilateralt lyskebrokopereret med smerter i begge sider

7. Metode

Patienter henvist til Tværfagligt Smertecenter, afsn. 7612, Rigshospitalet, med kroniske smerter efter lyskebrokkirurgi, og som opfylder inklusionskriterierne, vil få tilbud om at være med i undersøgelsen. Patienterne er henvist fra speciallæge (95 % via Dansk Herniedatabase). Kriterier for henvisning er at patienten har kraftige smerter efter lyskebrokoperationen med svær påvirkning af daglige funktioner. Foruden en standard formular for henvisning (Dansk Herniedatabases hjemmeside) skal der findes kopi af samtlige operationsbeskrivelser, notat fra seneste kliniske undersøgelse af lysken og svar på andre relevante undersøgelser (ultralyd, CT, MR, EMG). Ved den primære gennemgang optages anamnese og derefter foretages en detaljeret undersøgelse af lyskeområdet samt neurofysiologisk undersøgelse med QST. Patienterne inddeles i 2 grupper på baggrund af deres sensoriske profil i lyskeområdet: patienter med thermal hypoæstesi (≥ 3 øgede tærskler for [varmedetektionstærsklen [WDT], kuldedetektionstærsklen [CDT] og varmesmertetærsklen [HPT] eller kuldesmertetærsklen [CPT]]) vs. patienter med normo/hyperæstesi.

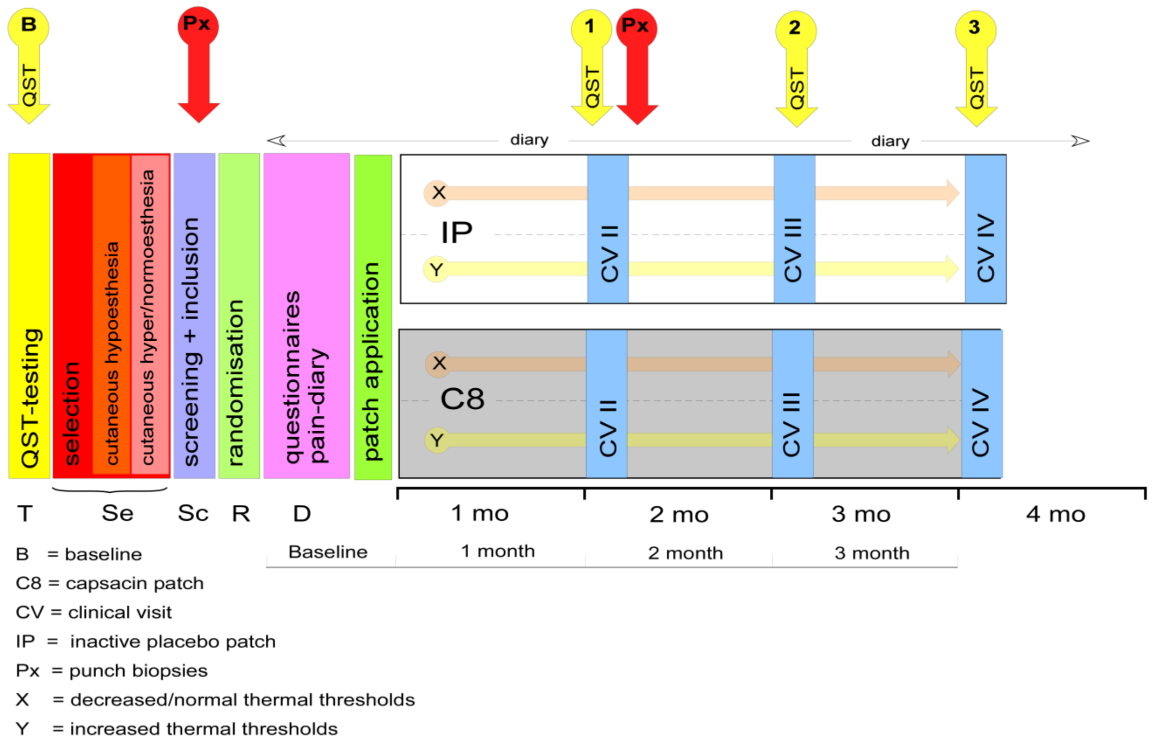

Forsøgsalgoritme(figur)

Figuren viser forsøgsalgoritmen der starter med initial kvantitativ sensorisk testning (T), selektion (Se) af patienter i 2 kategorier med kutan thermal hypæstesi eller thermal normo/hyperæstesi, screening og inklusion (Sc) hudbiopsi (Px) og blokrandomisering (R). Patienterne udfylder spørgeskemaer og smertedagbog ugen inden undersøgelsen starter(D). Efter applikationen af capsaicinplaster følges patienten ved 3 kliniske besøg med 1 måneds mellemrum hvor der også foretages QST. De kliniske besøg ligger på dag 30-36, dag 60-66 og dag 90-96. Der tillades en afvigelse i fremmøde fra det anførte på +/- 3 dage. Ved 1 måneds klinisk besøg (dag 30-36) tages desuden hudbiopsi.
Applikationen af plaster sker som anbefalet i Sundhedsstyrelsens produktresume og starter med forudbehandling af det markerede smertefulde hudområde med 4 % lidokain gel i en time. Herefter påsættes 1 plaster og plasteret forbliver på i 60 min. Smerter under og efter behandlingen behandles med lokal afkøling og orale analgetika (NSAID). En læge eller projektsygeplejerske vil være i kontakt med patienten mindst 1 gang hver 2. uge under opfølgningen for at afklare eventuelle spørgsmål, inklusiv mulige bivirkninger. Patienter vil når de afslutter eller evt. udgår fra undersøgelsen blive fulgt på vanlig vis på Rigshospitalets Tværfaglige Smertecenter.

7.a Smertescore og søvn

Smertescoring udføres i de sidste tre dage op til undersøgelsen starter, og i de sidste 3 dage op til hver af de tre kliniske besøg. Patienterne evaluerer 2 gange gang dagligt morgen og aften (for eksempel 08.00 og 20.00) smerteintensitet (NRS) i hvile, ved bevægelse (overgang fra liggende til stående stilling) og smerte ved palpation af mest smertefulde område i lysken. Desuden evalueres søvnkvaliteten ved et spørgsmål på en skala fra 0 til 10.[10]

7.b Kvantitativ sensorisk testning (QST)[5]

1. Deltagerne forberedes inden QST med trimning (operationstrimmer) af hårvækst i begge lysker og i det suprapubiske område. Derefter markeres med spritpen på begge sider tuberculum pubicum og spina iliaca anterior superior. Patienten indikerer med en finger punktum maksimum af lyskesmerterne som markeres med en cirkel. Et rektangulært måleområde på 2,5 x 5 cm^2 indtegnes rundt om punktum maksimum (cikatricen må ikke ligge i måleområdet).

2. Med en 25 ºC termoroller (Thermoroller, Somedic AB, Sverige) opspores og afgrænses områder med hypo- eller hyperæstesi (evt. allodyni) systematisk i lyskerne og suprapubisk. Man starter i normal hud og går radialt ind mod lyskeområdet i hver side. Når deltageren føler en ændring, siger han/hun til og der sættes et mærke med en spritpen. Arealer overføres efter QST til et eller flere transparente overheadark.
3. Derefter gøres termale tærskelbestemmelser med en 2,5 x 5,0 cm^2 termode som appliceres i måleområdet (MSA Thermotest, Somedic AB, Sverige). Der foretages målinger af varmedetektionstærsklen (WDT) og varmesmertetærsklen (HPT) hvor patienten ved hjælp af en trykknap skal afbryde varmestimuleringen ”lige præcis når temperaturen ændrer sig” og ”når varmen bliver til smerte” samt tilsvarende kuldedetektionstærsklen (CDT). Stimulationerne gentages 3 gange med 4-6 s randomiserede intervaller. Termoneutralitet er defineret ved 32,0 ºC og der bruges ved opvarmningen en ascenderende og descenderende ramp rate på respektive 1 og 5 ºC/s. Hvis patienten ikke trykker på knappen inden 50,0 ºC afbrydes varmestimuleringen og termoden går tilbage til 32,0 ºC.
4. Derefter foretages et kortvarigt tonisk varmestimulus (5 s, 47,0 ºC [STH = suprathreshold heat]) hvor patienten verbalt rater smerten via NRS (0-10). Stimulus starter på 32,0 ºC, der bruges en ramp rate på 1 ºC/s, når 47,0 ºC er nået holdes temperaturen i 5 s, hvorefter termoden går ned til udgangspunktet 32,0 ºC med en ramp rate på 1 ºC/s. Patienten kan på ethvert tidspunkt afbryde varmestimulus med trykknappen.
5. Endelig foretages smertetærskel måling med elektronisk trykalgometri (elektronisk trykalgometer, Somedic AB, Sverige). Trykalgometret består af en 1 cm^2 cirkulær probe der appliceres manuelt mod huden. Der appliceres et konstant stigende tryk 1-2 kPa/s og deltageren afbryder stimuleringen via en trykknap ”når trykket bliver smertefuldt” (PPT). Der foretages tre målinger med 30 s intervaller mellem hver måling. Hvis deltageren ikke har afbrudt stimuleringen ved 350 kPa stopper observatøren stimuleringen (”cut-off”).

QST undersøgelserne svarende til ovenstående er godkendt i de tidligere protokoller H-I-2010-116 og H-2-2011-051.

7.c Hudbiopsier

Med henblik på bestemmelse af intraepidermal nerve fiber densitet (IENFD), tages hudbiopsi inden behandlingsstart og ved 1 måneds klinisk kontrol, med 3 mm stans (Disposable biopsy punch, WPI,

USA) under sterile forhold, efter lokalbedøvelse med 1 % mepivacain s.c.[11;12] Der udtages 1 hudbiopsi på den kontralaterale og 1 hudbiopsi på den opererede side indenfor 12,5 cm^2 måleområderne. Biopsierne fikseres i 4 % formalin i 24 timer, vaskes i fosfat buffer og opbevares i 10 % sucrose med 0,1 M fosfatbuffer. Prøverne sendes straks herefter direkte til Prof. Dr. Claudia Sommer og medarbejdere, Neurologische Klinik, Universitätsklinikum, Würzburg, Tyskland med henblik på analyse. I en kryostat skæres 50 µm snit der via dobbelimmunofluorescens teknik interagerer med panneuronal marker PGP9.5 (1:800, Ultraclone, UK) og visualiseres med Cy3-mærket anti-kanin antistof (1:100, Amersham, USA). IENFD bestemmelsen sker med Zeiss Axiophot 2 microscope (Wetzlar, Germany) and Spot advanced software (Windows Vers. 4.5). Observatøren der foretager IENFD beregningerne er blindet for tilhørsforholdet af biopsimaterialet.[11] Biopsimaterialet destrueres straks efter analysen.

7d. Randomisering og blinding

Randomiseringen vil blive foretaget af Herning Apotek der også vil stå for pakning og etikettering af capsaicinplaster og placeboplaster efter givne regler. Der foretages blokrandomisering af 4 patienter. Der udarbejdes 2 sæt forseglede kodekuverter. Disse indeholder oplysning om, hvilken behandling den enkelte patient er randomiseret til. Det ene sæt opbevares af sponsor, det andet opbevares af koordinerende investigator på et sikkert aflåst sted. Der vil være en procedure for akut afblinding af patienter i form af en forseglet kuvert som medfølger hver pakning med patient nummer med angivelse af den aktuelle behandling. Apoteket opbevarer den afblindede randomiseringsliste, som først vil blive tilsendt investigator, når dataanalyse er foretaget. Blindingen må kun brydes, hvis den fortsatte behandling af patienten nødvendiggør kendskab til randomiseringskoden. Hvis kodekuverten åbnes, skal dato og årsag registreres, og kuverten skal signeres af investigator. Dette medfører eksklusion af patienten. Dobbeltblindingen i forsøget sikres ved at det er en af forsøget uafhængig sygeplejerske/læge der vil stå for påsætning af plaster. Capsaicinplaster og placebo plaster er identiske i udseende.

7 e. Undersøgelsesmedicin:

<u>Aktivt præparat</u>:

Qutenza 179 mg kutanplaster

Hvert kutanplaster pa 280 cm^2 indeholder i alt 179 mg capsaicin svarende til 640 mikrogram capsaicin pr. cm^2 plaster (8 % w/w).

Indehaver af markedsføringstilladelsen er:
Astellas Pharma Europe B.V. Elisabethhof 19, 2353 EW Leiderdorp, Holland.

Aktivt placebo præparat:
Placeboplaster udviklet af Astellas. Placeboplasteret er identisk i udseende med det aktive capsaicinplaster hvilket sikrer blinding.

Investigator vil føre medicinregnskab og tilse, at capsaicinplastre og placeboplastre opbevares på et sikkert sted. Investigator gør rede for plastre, som ved et uheld eller på anden måde er bortkommet. Patienten må indtage per orale smertestillende midler under studiet forudsat af at behandlingen har været stabil i mindst 4 uger før forsøgsstart. ”Rescue medicin” under undersøgelsen i form af tablet paracetamol (højest 4 g i døgnet) er dog tilladt og forbrug registreres.

8. Outcome

8.a Outcome, patienter

8.a.1 Primære

Den summerede smerteintensitet - summed pain intensity (SPI) udregnes som median smerteintensitet i hvile, i bevægelse og ved autopalpation af PM to gange dagligt i de sidste 3 dage af hver studie fase: Baseline ($0SPI_{3d}$), Måned 1 ($1SPI_{3d}$), Måned 2 ($2SPI_{3d}$) Måned 3 ($3SPI_{3d}$). Differencen i summeret smerteintensitet - Summed pain intensity differences (SPID) udregnes:

- $0SPI_{3d}$ - $1SPI_{3d}$ | = $SPID_{0-1}$
- $0SPI_{3d}$ - $2SPI_{3d}$ | = $SPID_{0-2}$
- $0SPI_{3d}$ - $3SPI_{3d}$ | = $SPID_{0-3}$

Primære outcome er maksimum differencen i summeret smerteintensitet (SPID) og Δ SPID udregnes som differencen i SPID mellem patienter behandlet med capsaicinplaster og placeboplaster. Tilsvarende udregninger laves for undergrupperne: patienter med thermal hypoestesi og thermal normo/hyperestesi.

8.a.2 Sekundære

- Spørgeskemaer: Activities Assessment Scale (AAS)[4] der er et spørgeskema til vurdering af smerterelateret funktionsnedsættelse. Hospital Anxiety and Depression Scale (HADS)[13] og Pain Catastrophizing Scale (PCS)[14] til vurdering af psykologiske faktorer og Leeds self-assessment of neuropathic symptoms and signs (S-LANSS)[15] til vurdering af neuropatiske smerter. Desuden evalueres søvnkvalitet.[10]
- Forbrug af yderlig smertestillende medicin (paracetamol)
- IENFD (△ opereret side – kontralateral side)

9. Statistik

Power beregningen hviler på forudsætningerne:

- at data er normalfordelt
- at SD for 0SPI3d målingerne (6 målinger [2 gange dagligt i 3 dage] giver en intraindividuel range på 30 – 42 (NRS) svarer til SD = 3,1 (gennemsnitlig initial smertescoring er 6 NRS (0-10) med range 5 – 7. 6 målinger giver 30-42 NRS and derfor 3,92 x SD = 12, hvilket giver 1 SD = 3.1)
- at den minimale relevante difference sættes til gennemsnitlig 4.5 i NRS score (hvilket svarer til en ændring i ΔSPID > 8 %)
- at α sættes til 0,01
- at β sættes til 0,1 (power = 0,9)
- at designet er et parallel gruppe studie

Antallet af patienter der skal bruges i hver gruppe bliver herved 24 (total 48 [MedCalc® Software 11.5.1.0]). På grund af en vis usikkerhed ved power beregningen foretages efter inklusion af 32 evaluerbare patienter en interim analyse af ekstern statistiker. Studiet stoppes hvis der findes signifikant forskel mellem aktivt behandlede og placebo behandlede patienter på 0,01 signifikansniveau eller hvis en beregning viser at inklusion af det maksimalt tilladte antal patienter (n = 50) ikke vil kunne give den forventede statistiske forskel mellem de to grupper. Der kan inkluderes yderligere patienter op til maksimalt 25 i hver gruppe (total 50 patienter).

Fordelingen af kontinuerlige data bedømmes for normalitet med Shapiro-Wilk W test (SPSS 18.0, Chicago, Illinois). Statistisk sammenligning af parrede data gøres med parametriske eller non-parametriske metoder, afhængig af fordelingen. Som beskrevet bruges SPID, et summations mål, dels for at mindske risikoen for massesignifikans ved gentagne målinger, dels for at undgå alt for

konservative korrektioner (Bonferroni's test), hvilket øger risikoen for type II fejl. Ved statistiske sammenligninger mellem kategoriske variabler bruges Fischers exact test.
Datamaterialet gennemgås både for ITT (intention-to-treat) og PP (per-protocol). Ved ændring af den statistiske plan vil der blive redegjort herfor ved publicering. Ekskluderede patienter samt manglende, ubenyttede eller uægte data vil blive beskrevet.

10. Ulemper for patienten

En af ulemperne vil bestå i at patienten under QST-målingerne er afklædt i lyske området og at en barbering af hårvækst i dette område kan være nødvendig. Deltagerne har dog hele tiden kønsorganerne dækket til. Undersøgelsen foregår i et tempereret og ugeneret rum. En anden ulempe er de smertefremkaldende hudpåvirkninger ved QST-måling, som dog er meget kortvarige. Sandsynligheden for hudskader er minimal. Patienten kan selv til hver tid afbryde stimuleringerne ved at trykke på en knap.
En tredje ulempe er det ubehag som hudbiopsierne kan fremkalde. Den 3 mm cirkulære stansebiopsi er overfladisk og sjældent associeret med smerte.[12] Der lægges forinden lokalbedøvelse med mepivacain 1 % for at forebygge ubehaget. Der efterlades et 3 mm overfladisk sår som læges indenfor 1-2 uger.

11. Bivirkninger og komplikationer.

Der er ingen bivirkninger til QST undersøgelsen. Der findes en meget lille risiko for at der kan opstå overfladisk infektion i såret efter hudbiopsien selvom steril teknik anvendes.
Bivirkninger til capsaicinplasteret ses ifølge produktresume fra Sundhedsstyrelsen hyppigst i form af lokaliserede hudreaktioner efter plaster påsættelse. De hyppigste er smerte på applikationsstedet samt erytem, pruritus, papler, vesikler, ødemer, hævelser og tørhed pa applikationsstedet. Den systemiske eksponering for capsaicin er meget lav.
Registrering af bivirkninger starter ved påsætningen af plaster og stopper ved patientens sidste besøg. Sponsor skal af investigator løbende holdes orienteret om bivirkninger og hændelser. Investigator skal øjeblikkeligt informere sponsor om alvorlige hændelser og bivirkninger (som defineret i Sundhedsstyrelsens vejledning). Ved forsøgets afslutning skal den endelige rapport til Sundhedsstyrelsen indeholde en beskrivelse af alle indtrufne bivirkninger og hændelser. Serious adverse reaction (SAR) (Alvorlige, formodede bivirkninger), skal af sponsor indberettes én gang årligt til Sundhedsstyrelsen. Indberetningen skal også indeholde en rapport over forsøgspersoners

sikkerhed. Suspected unexpected serious adverse reaction (SUSAR) (Uventede og alvorlige formodede bivirkninger), som er dødelige eller livstruende skal hurtigst muligt og senest 7 dage efter at sponsor har fået kendskab til en sådan formodet bivirkning indberettes til Sundhedsstyrelsen og senest 8 dage efter indberetningen skal sponsor meddele Sundhedsstyrelsen alle relevante oplysninger om opfølgningen på indberetningen. Sundhedsstyrelsens produktresume for capsaicin plaster anvendes som referencedokument, ved vurdering af om en alvorlig relateret bivirkning er uventet eller ventet.
Alle andre uventede og alvorlige formodede bivirkninger, skal indberettes til Sundhedsstyrelsen senest 15 dage efter, at sponsor har fået kendskab til disse. Enhver indberetning skal ledsages af kommentarer om evt. konsekvenser for forsøget.

12. Respekt for deltagernes integritet
Alle love og regler vil blive overholdt og data registreret i forbindelse med undersøgelsen og bliver anmeldt til Datatilsynet (j.nr. XXX). Deltagere i undersøgelsen beskyttes efter lov om behandling af personoplysninger og sundhedsloven. Undersøgelsen vil blive anmeldt til Clinicaltrials.gov.

13. Dataregistrering
Alle personrelaterede data behandles fortroligt. Alle oplysninger vil blive behandlet fortroligt og ved indberetning af forsøgsresultater vil patienterne være anonyme og de ansvarlige personer for dette forsøg er underlagt tavshedspligt. Investigator vil føre en identifikationsliste over alle patienter, som har fået tildelt forsøgsnumre. Denne liste vil indeholde patienternes fulde navn og CPR-nummer. Indsamlede data i form af Case Report Form og journal vil kun blive gjort tilgængelige for tredje part i overensstemmelse med dansk lovgivning. Dette vil sige i forbindelse med monitorering fra Københavns Universitets Hospitals GCP (Good Clinical Practice) enhed. Herudover ved inspektion af autoriserede repræsentanter fra relevante myndigheder herunder Sundhedsstyrelsen. Patienterne vil skriftligt blive informeret om, at resultaterne vil blive opbevaret og analyseret i en computer, at patienternes anonymitet vil blive bevaret og at den lokale datalovgivning vil blive overholdt. Data vil blive opbevaret efter datatilsynets regler. Patienterne vil også skriftligt blive informeret om muligheden for revision fra offentlige myndigheder i det tilfælde, at disse kræver indsigt i relevante oplysninger, samt at Københavns Universitets Hospitals GCP Enhed også vil have adgang til disse oplysninger. Investigator sikrer at projektet vil følge regler for ICH-GCP.

14. Kvalitetskontrol og kvalitetssikring

Skulle Datatilsynet, Sundhedsstyrelsen, GCP-enheden eller andre relevante myndigheder ønske adgang til kildedata eller andre forsøgsrelaterede dokumenter vil disse få direkte adgang. God klinisk praksis praktiseres for at sikre, at forsøgspersonernes rettigheder, sikkerhed og velfærd beskyttes, og at data fra de kliniske forsøg er troværdige. Der vil på forsøgscenteret være CRFs, som indeholder information om hver enkel forsøgsperson, og data opsamlet under forsøget. Endvidere vil almindelige procedurer for kvalitetskontrol og kvalitetssikring følges. En monitor tilknyttet GCP-enheden, København, vil være ansvarlig for at monitorere forsøget. Den ansvarlige monitor vil kontakte og aflægge besøg på forsøgscenteret, og vedkommende vil efter anmodning få tilladelse til at inspicere de forskellige forsøgsrelaterede dokumenter. Det er den koordinerende investigator og forsøgsansvarliges ansvar, at eventuelle fejl og mangler, der bliver opdaget under monitoreringen bliver udbedret.

15. Indhentelse af samtykke

Ved information af patienten på den primære undersøgelsesdag, som foregår i et stille lokale med lukket dør, oplyser den lægelige undersøger (JB, MW) at det drejer sig om et videnskabeligt forsøg, at patienten på ethvert tidspunkt har ret til at afstå fra forsøget, uden at det vil påvirke eventuelle nuværende og fremtidige behandlinger. Patienten oplyses op retten til at have en bisidder med. Ønskes dette aftales et nyt samtaletidspunkt. Vi oplyser om at undersøgelsen er godkendt af Videnskabsetisk Komité, Sundhedsstyrelsen og Datatilsynet. Strukturen af informationssamtalen fremgår af videnskabsetisk komites ”retningslinjer for afgivelse af mundtlig deltagerinformation”. Efter den mundtlige orientering gennemlæser patienten den skriftlige information, som også indeholder ”Forsøgspersonens rettigheder i et biomedicinsk forskningsprojekt.” Herefter spørges om patienten har spørgsmål til undersøgelsen. Undersøgeren redegør derefter for hypotese og metode, risici, bivirkninger, komplikationer, samt fordele og ulemper for patienten. Patienten oplyses igen om retten til at ophæve sit informerede samtykke når som helst uden forklaring. Patienten informeres om muligheden for at få indblik i undersøgelsens resultater. Samtalen afsluttes med mulighed for yderligere spørgsmål. Patienten har herefter en betænkningstid på 24 timer hvorefter undersøgende læge kontakter patienten telefonisk for at høre om der er yderligere spørgsmål og om patienten samtykker i deltagelse. Den underskrevne samtykkeerklæring afleveres på forsøgsdagen hvilket vil være 2-14 dage efter samtalen.

16. Økonomiske forhold og forsikring

Plastrer (capsaicin 8 % og placebo), transportomkostninger for patienter samt aflønning af projektsygeplejerske under studiet er betalt af Astellas Pharma a/s Danmark, som giver støtte til ovennævnte ting for i alt 750.000 kr. Forsøgsansvarlige og øvrige investigatorer har ingen økonomisk tilknytning til Astellas. Hospitalsudstyr, undersøgelsesfaciliteter og kontor/kontorfaciliteter stilles til rådighed af Enhed for Kirurgisk Patofysiologi, afsnit 4114 og Tværfagligt Smertecenter, afsnit 7612, Rigshospitalet. Initiativtager til undersøgelsen er Joakim Bischoff, Enhed for Kirurgisk Patofysiologi/Tværfagligt Smertecenter og Mads Werner, Tværfagligt Smertecenter, Rigshospitalet. Patienterne der indgår i undersøgelsen er dækket af Patientforsikringen. (http://www.patientforsikringen.dk/).

17. Etik

Undersøgelsen ligger i forlængelse af tidligere arbejder omhandlende langvarige smerter efter kirurgi. Forsøget udføres i overensstemmelse med protokollen og gældende myndighedskrav og lovgivning. Deltagelse i den kliniske undersøgelse vil først ske efter indhentelse af informeret og accepteret samtykke. Valide kliniske undersøgelser betjener sig af et dobbeltblind, randomiseret og placebokontrolleret design. Målingerne (QST) er de normalt forekommende i Tværfagligt Smertecenters regi. Det kan give anledning til etiske overvejelser når den ene halvdel af patienter i en undersøgelse modtager placebo. Vi mener dog ikke at der er etiske betænkeligheder i denne undersøgelse da alle patienter der indgår, må tage deres normale faste smertestillende medicin, således at selv de patienter der modtager placeboplaster ikke er stillet dårlige ved at indgå i undersøgelsen. Undersøgelsens resultater vil muligvis kunne resultere i en ny lokal behandling, med lav risiko for systemiske bivirkninger, til patienter med kroniske smerter efter lyskebrokoperation. Hudbiopsi med bestemmelse af nervefiberdensitet (IENFD) vil øge forståelsen af de patofysiologiske mekanismer bag smerterne og hvis det viser sig at patienter der responderer på behandlingen med capsaicinplaster har en specifik IENFD profil vil det kunne bruges fremadrettet ved udvælgelse af patienter til behandling med capsaicinplaster. Erfaringerne fra dette studie vil formentlig ligeledes kunne benyttes i behandlingen af kroniske smerter efter andre former for kirurgi for eksempel kroniske smerter efter thoraxkirurgi og brystcancerkirurgi. Ansvarlige investigator er ansvarlig for at informere den videnskabsetiske komité om enhver alvorlig utilsigtet

hændelse og/eller større ændringer i protokollen. Al korrespondance arkiveres af den koordinerende investigator.

18. Publikation

Både positive og negative og inkonklusive resultater vil blive offentliggjort. Studiets resultater vil om muligt blive publiceret i et internationalt videnskabeligt tidsskrift. Forfatterrækkefølgen følger International Committee of Medical Journal Editors (IMCJE)-reglerne[1] og er:

Bischoff JM, Ringsted TK, Kehlet H, Werner MU med corresponding author Bischoff JM.

[1] http://www.icmje.org/ethical_1author.html

Referenceliste

1. Kehlet H: Chronic pain after groin hernia repair. Br.J.Surg. 2008; 95: 135-6
2. Aasvang EK, Gmaehle E, Hansen JB, Gmaehle B, Forman JL, Schwarz J, Bittner R, Kehlet H: Predictive risk factors for persistent postherniotomy pain. Anesthesiology 2010; 112: 957-69
3. Aasvang EK, Kehlet H: The effect of mesh removal and selective neurectomy on persistent postherniotomy pain. Ann.Surg. 2009; 249: 327-34
4. McCarthy M, Jr., Jonasson O, Chang CH, Pickard AS, Giobbie-Hurder A, Gibbs J, Edelman P, Fitzgibbons R, Neumayer L: Assessment of patient functional status after surgery. J.Am.Coll.Surg. 2005; 201: 171-8
5. Aasvang EK, Brandsborg B, Jensen TS, Kehlet H: Heterogeneous sensory processing in persistent postherniotomy pain. Pain 2010; 150: 237-42
6. Wasner G, Kleinert A, Binder A, Schattschneider J, Baron R: Postherpetic neuralgia: topical lidocaine is effective in nociceptor-deprived skin. J.Neurol. 2005; 252: 677-86
7. Haanpaa M, Attal N, Backonja M, Baron R, Bennett M, Bouhassira D, Cruccu G, Hansson P, Haythornthwaite JA, Iannetti GD, Jensen TS, Kauppila T, Nurmikko TJ, Rice AS, Rowbotham M, Serra J, Sommer C, Smith BH, Treede RD: NeuPSIG guidelines on neuropathic pain assessment. Pain 2011; 152: 14-27
8. Geber C, Birklein F: Dissecting post-herniotomy pain--scratching the surface? Pain 2010; 150: 215-6
9. Galer BS: Topical analgesic medication - the dawn of a new era. Pain 2009; 147: 5-6
10. Vernon MK, Brandenburg NA, Alvir JM, Griesing T, Revicki DA: Reliability, validity, and responsiveness of the daily sleep interference scale among diabetic peripheral neuropathy and postherpetic neuralgia patients. J.Pain Symptom.Manage. 2008; 36: 54-68
11. Torvin MA, Winther BF, Feldt-Rasmussen U, Rasmussen A, Hasholt L, Lan H, Sommer C, Kolvraa S, Ballegaard M, Staehelin JT: Functional and structural nerve fiber findings in heterozygote patients with Fabry disease. Pain 2009; 145: 237-45
12. Walk D: Role of skin biopsy in the diagnosis of peripheral neuropathic pain. Curr Pain Headache Rep. 2009; 13: 191-6
13. Zigmond AS, Snaith RP: The hospital anxiety and depression scale. Acta Psychiatr.Scand 1983; 67: 361-70
14. Sullivan MJL, Bishop SR, Kivik J: The pain catastrophizing scale: development and validation. Psych Assess 1995; 7: 524-32

15. Bennett MI, Smith BH, Torrance N, Potter J: The S-LANSS score for identifying pain of predominantly neuropathic origin: validation for use in clinical and postal research. J.Pain 2005; 6: 149-58
